# *Helicobacter pylori* Infection of Primary Human Monocytes Boosts Subsequent Immune Responses to LPS

**DOI:** 10.3389/fimmu.2022.847958

**Published:** 2022-03-02

**Authors:** Tobias Frauenlob, Theresa Neuper, Muamera Mehinagic, Hieu-Hoa Dang, Diana Boraschi, Jutta Horejs-Hoeck

**Affiliations:** ^1^ Department of Biosciences, University of Salzburg, Salzburg, Austria; ^2^ Cancer Cluster Salzburg (CCS), Salzburg, Austria; ^3^ Institute of Biochemistry and Cell Biology (IBBC), National Research Council (CNR), Napoli, Italy; ^4^ Department of Biology and Evolution of Marine Organisms, Napoli, Italy; ^5^ Shenzhen Institute of Advanced Technology (SIAT), Chinese Academy of Sciences (CAS), Shenzhen, China

**Keywords:** monocytes, innate memory, innate immunity, *Helicobacter pylori*, chronic gastric inflammation, primary myeloid immune cells, Endotoxin tolerance

## Abstract

Infection with *Helicobacter pylori (H. pylori)* affects almost half of the world’s population and is a major cause of stomach cancer. Although immune cells react strongly to this gastric bacterium, *H. pylori* is still one of the rare pathogens that can evade elimination by the host and cause chronic inflammation. In the present study, we characterized the inflammatory response of primary human monocytes to repeated *H. pylori* infection and their responsiveness to an ensuing bacterial stimulus. We show that, although repeated stimulations with *H. pylori* do not result in an enhanced response, *H. pylori*-primed monocytes are hyper-responsive to an *Escherichia coli*-lipopolysaccharide (LPS) stimulation that takes place shortly after infection. This hyper-responsiveness to bacterial stimuli is observed upon infection with viable *H. pylori* only, while heat-killed *H. pylori* fails to boost both cytokine secretion and STAT activation in response to LPS. When the secondary challenge occurs several days after the primary infection with live bacteria, *H. pylori-*infected monocytes lose their hyper-responsiveness. The observation that *H. pylori* makes primary human monocytes more susceptible to subsequent/overlapping stimuli provides an important basis to better understand how *H. pylori* can maintain chronic inflammation and thus contribute to gastric cancer progression.

## Introduction

*H. pylori*, a stomach-residing pathogen, is considered to be one of the main causative agents of stomach neoplasms, and has been classified as a class I carcinogen by the World Health Organization (1). Following *H. pylori* infection, both epithelial cells and immune cells initiate physical, chemical and biological processes to defend the organism against pathogenic colonization. However, the pathogen has developed sophisticated strategies to dampen immune responses and thus establish long-term infection. Persistent infection may cause chronic inflammation, which leads to severe disruption of homeostasis in the stomach and can be a major cause of gastric ulcers and gastric neoplasms, including gastric adenocarcinoma and lymphoma (2). Immune cells that are activated during colonization of the stomach lining by *H. pylori* significantly contribute to the development of a chronically inflamed environment (3, 4). Attracted by mucosa-originating chemokines, cells such as neutrophils, monocytes, dendritic cells and T cells migrate to the infection sites, where they are activated by *H. pylori* or its pathogen-associated molecular patterns (PAMPs) (5–7). Monocytes, in particular, are potent producers of inflammation-driving cytokines associated with gastric cancer development, among them tumor necrosis factor (TNF)-α, interleukin (IL)-1β and chemokine (C-X-C motif) ligand (CXCL) 8 (8–11). Other secreted mediators, such as IL-12p70, favor the generation of type 1 T helper responses and may further exacerbate tissue injury (12, 13). Despite this increased secretory activity and subsequent recruitment of vast numbers of leukocytes, including monocytes, to the infected epithelium (14, 15), the host is rarely able to clear *H. pylori*, resulting in chronic infection. Long-term colonization of the gastric mucosa is further supported by the induction of tolerogenic antigen-presenting cells and the subsequent generation of regulatory T cells (16–18). The resulting dysregulated inflammatory processes can provide fertile ground for epithelial de-differentiation. Atrophy of parietal cells, for instance, may occur as a consequence of chronic inflammation (19), leading to low gastric acid levels, which in turn favor bacterial overgrowth (20), potentially further increasing inflammatory responses.

Since uncontrolled immune responses and associated aberrant inflammatory processes are crucial drivers of gastric adenocarcinoma development, immune cell activation needs to be tightly regulated. Innate immune cells, including monocytes, have developed intricate mechanisms to limit production of inflammatory mediators in response to microbial stimuli (21–24). In the 1940s, the term endotoxin tolerance was coined to describe reduced fever following repeated intravenous injections of typhoid toxins (25). Since then, the molecular mechanisms underlying the phenomenon by which monocytes stimulated with normal to high doses of lipopolysaccharide (LPS) (ng-µg/mL) become incapable of reacting to a second LPS stimulus have been extensively studied (26, 27). Limiting the response to a second bacterial stimulus is a protective measure sparing infected tissue from damage. However, initial treatment with very a low dose of LPS, which does not induce inflammatory cytokine production results in augmented expression of inflammatory mediators upon a second high-dose LPS challenge (28).

As already mentioned, the mechanisms behind the chronicity of *H. pylori* infections remain incompletely understood. It is likely that the innate immune system contributes to the inflammatory environment and subsequent chronic inflammatory processes following pathogen recognition, although information on monocyte activity upon repeated encounters with *H. pylori* is scarce. Therefore, we aimed at elucidating whether infection with the pathogen renders immune cells less responsive to a subsequent stimulus, as previously observed with LPS, or if *H. pylori* may circumvent this host-protection mechanism.

## Material and Methods

This study was conducted in agreement with the guidelines of the World Medical Association’s Declaration of Helsinki. Given that national regulations do not require informed consent in the case of anonymous blood cells that are discarded after plasmapheresis (buffy coats), no additional approval by the local ethics committee was required.

### Isolation of Monocytes

Monocytes were isolated from fresh buffy coats from healthy anonymous donors, provided by the Blood Bank Salzburg. Peripheral blood mononuclear cells (PBMCs) were obtained by gradient density separation with Histopaque-1077 (Sigma-Aldrich, Vienna, Austria). Monocytes were further purified *via* magnetic labeling using CD14 MicroBeads (Miltenyi Biotec, Bergisch-Gladbach, Germany) according to the manufacturer’s instructions. Monocytes were then cultured in RPMI-1640 medium (Sigma-Aldrich) supplemented with 10% heat-inactivated fetal calf serum (FCS; Biowest, Nuaillé, France) and 1% L-glutamine (Fisher Scientific, Schwerten, Germany). Generally, monocytes were rested between 1 and 4 h after isolation prior to being subjected to further treatment.

### Bacterial Culture and Infection

*H. pylori* P12 wild type (Hp) was cultured in a bacterial culture Oxoid 3.5 L bucket (Thermo Fischer Scientific, Vienna, Austria) under microaerophilic conditions generated by using an Oxoid CampyGen 3.5 L Sachet (Thermo Fisher Scientific), and at 37°C on GC agar plates containing 10% horse serum (Biowest, Vienna, Austria). For infection, bacterial cells were harvested from culture plates using cotton buds (Paul Boettger, Bodenmais, Germany) and solubilized in 1 mL PBS (Sigma-Aldrich). Optical density of the solution was determined by spectrophotometric measurement (OD600) on a BioPhotometer Plus device (Eppendorf, Vienna, Austria) and the bacterial cell count was calculated against an in-house calibration curve. The density of bacterial cells was then adjusted according to the experimental requirements. Multiplicities of infection (MOI) of 5, 1 and 0.2 were used.

### Monocyte Short-Term Innate Response Model

Monocytes were exposed to stimuli on two consecutive days (**Figure 2A**). On day 1, 1×10^5^ monocytes were seeded in 100 µL of cell culture medium (RPMI-1640, 10% heat-inactivated FCS, 1% L-glutamine) in 96-well flat-bottom cell culture plates and rested for 1-4 h at 37°C. Then, 100 µL of cell culture medium containing either *E. coli* LPS (O55:B5; final concentration of 5 ng/mL), live bacteria (*H. pylori* P12) or heat-killed *H. pylori* cells at MOI 5 were added to each well to a final volume of 200 µL, and incubated for 24 h at 37°C and 5% CO_2_. On day 2, supernatants were carefully removed from each well and stored, leaving the adherent monocytes unperturbed at the bottom of the well. Immediately after, 200 µL of fresh medium containing 1% Pen/Strep and either 10 ng/mL final concentration of LPS (challenge), *H. pylori* (MOI5) or medium only were added. The cells were then cultured for a further 24 h, and supernatants collected for analysis. For RNA and protein expression studies, this second incubation period was terminated 2 h post-challenge.

### Monocyte Long-Term Innate Response Model

This experimental setup is based on an *in vitro* model of innate memory, published by Madej et al. (29). Briefly, monocytes were incubated for 7 days between the two treatments (**Figure 5A**). Similar to the short-term innate response model, monocytes were seeded on day 1 at a density of 1×10^5^ cells in RPMI- 1640 medium containing 5% heat-inactivated human serum type AB (Lonza, Basel, Switzerland) and 1% L-glutamine, and primed with *H. pylori* at different MOIs (0.2, 1 or 5) for 24 h. On day 2, supernatants were carefully removed and stored, and fresh medium containing 1% Pen/Strep was added to the wells. Cells were then cultured for 6 days, with a change of medium at day 4. On day 8, medium was removed and fresh medium was added to the wells, either containing 5 ng/mL LPS (challenge) or not (control). Monocytes where then cultured for an additional 24 h, and on day 9 the supernatants were collected for analysis.

### ELISA

TNF-α and IL-6 ELISAs (Peprotech, London, United Kingdom) were performed on supernatants harvested from experimentally treated human monocytes, according to the manufacturer’s instructions.

### Multiplex-Based Detection of Secreted Mediators

Inflammatory mediator production and secretion by monocytes into the supernatant was measured by Multiplex bead-based assay using the Cytokine/Chemokine/Growth Factor 45-Plex Human ProcartaPlex Panel or the Inflammation 20-Plex Human ProcartaPlex Panel (Thermo Fisher Scientific). Briefly, mediator-binding beads were washed once (PBS, 0.05% Tween-20), resuspended in Assay Buffer and distributed in wells of a 96-well V-bottom plate. Aliquots of standards or samples (15 µL) were added, and the plate was incubated on an orbital shaker at 4°C in the dark overnight. The plate was then washed three times with 150 µL/well wash buffer and incubated with 15 µL/well of Detection Antibody solution on an orbital shaker for 30 min at room temperature (RT). The plate was again washed three times and incubated for another 30 min with 20 µL/well of Streptavidin-PE solution. After washing (3x as above), Reading Buffer was added to the wells for analysis. Quantification was performed on a Luminex MagPix instrument (Luminex, Austin, TX, USA), and data were analyzed with ProcartaPlex Analyst software (Thermo Fisher Scientific).

### mRNA Expression Analysis

Total cellular RNA was extracted with TRI Reagent (Sigma-Aldrich) according to the manufacturer’s instructions. Pure RNA was then reverse transcribed utilizing RevertAid H Minus M-MuLV reverse transcriptase (Thermo Fisher Scientific). Expression levels of genes of interest were determined by quantitative real-time PCR on a Rotor Gene 3000 instrument (Corbett Research, Cambridge, UK), using Luna Universal qPCR Master Mix (New England BioLabs, Ipswich, MA, USA). mRNA expression was normalized to the expression of a housekeeping gene (RPLP0). Relative mRNA expression was calculated as 2^-ΔCt^, where the ΔCt value represents the difference between the threshold cycle (Ct) of the gene of interest minus the threshold cycle of the housekeeping gene. Specificity of primers was monitored *via* analysis of product melting curves. The following primer pairs were used: *TNF* fwd: 5’- caa gcc tgt agc cca tgt tg -3’, *TNF* rev: 5’- gag gtt gac ctt ggt ctg gta -3’*, IL12A* fwd: 5’- tca gca tgt gtc cag cgc gca -3*’, IL12A* rev: 5’- tct ctt cag aag tgc aag gg -3’, *IL6* fwd: 5’- gta cat cct cga cgg cat ctc -3’*, IL6* rev: 5’- ggc aag tct cct cat tga atc -3’*, IL10* fwd: 5’- agg gca ccc agt ctg aga aca -3’*, IL10* rev: 5’- cgg cct tgc tct tgt ttt cac -3’.

### Flow Cytometry

Cell surface expression of co-stimulatory molecules on pure monocytes was determined by analysis of median fluorescence intensity on a FACS Canto II flow cytometer (BD Biosciences, San Francisco, CA, USA). Cells were seeded in V-bottom plates after isolation and then cultured with the indicated priming stimulus for 24 h, after which the cells were centrifuged and the supernatants discarded. Thereafter, cells were subjected to the indicated challenge stimulus for 20 h. Cells were then washed in PBS before being stained in 30 µL of staining mix for 30 min in the dark at 4°C. Light exposure was minimized throughout to avoid photobleaching. After staining, the cells were washed and fixed with 4% paraformaldehyde for 15 min at 4°C, then washed twice and resuspended in PBS + 2 mM EDTA for analysis. The following antibody-fluorophore conjugates were used: CD14 PerCP Cy-5.5 (MϕP9, BD Biosciences), Fixable Viability Dye eFluor 506 (eBioscience Thermo Fisher Scientific), CD40-FITC (5C3; eBioscience Thermo Fisher Scientific), CD83-PE-Cy7 (HB15e, BD Biosciences). Data analysis was performed using FlowJo 10 software.

### Western Blot Analysis

Cell lysates were prepared in NP40 sample buffer, containing 150 mM NaCl, 1% Triton X, 50 mM Tris pH 8.0, 1 mM PMSF (protease inhibitor) and phosphatase inhibitor, diluted 1:1 with 2X Laemmli sample buffer (Bio-Rad, Vienna, Austria) containing 5% beta-mercaptoethanol. Samples were then separated on a 4-12% NuPAGE Bis-Tris gel (Invitrogen, Vienna, Austria) and transferred onto a nitrocellulose membrane (0.45 µm). After blocking of non-specific binding sites with 5% skim milk for 1 h at RT under gentle agitation, the membrane was incubated with the appropriate antibody, prepared in 5% bovine serum albumin diluted in Tris-buffered saline containing 0.1% TWEEN20 (TBS-T) or 5% skim milk in TBS-T, overnight at 4°C under gentle agitation. The membrane was then washed and incubated with the appropriate secondary antibody conjugated with horseradish peroxidase (HRP) for 1 h at RT under gentle agitation. After washing, the membrane was incubated with West Pico PLUS chemiluminescent substrate (Thermo Fisher Scientific), and detection was performed with a ChemiDoc Imager (Bio-Rad). The following primary and secondary antibodies were used according to the manufacturer’s instructions: phospho-STAT1 (Tyr701) (58D6) rabbit mAb #9167, phospho-STAT3 (Tyr705) (D3A7) XP rabbit mAb #9145, STAT1 (D4Y6Z) rabbit mAb #14995, STAT3 (D3Z2G) rabbit mAb #12640 (all from Cell Signaling Technology, Frankfurt, Germany).

### Statistical Analysis

Data are represented as symbols indicating mean ± standard deviation (SD) or as bars indicating mean + SD. Statistical analyses were performed *via* GraphPad Prism 9 Software (GraphPad Software, San Diego, CA, USA). Differences between multiple stimulation groups were analyzed *via* two-way ANOVA or matched/repeated measures one-way ANOVA including appropriate *post-hoc* tests. Sample size is indicated in the figure legends. *p* values <0.05 were considered significant (*p ≤ 0.05, **p ≤ 0.01, ***p ≤ 0.001, ****p ≤ 0.001).

## Results

### Potent Secretion of Cytokines and Chemokines Upon *H. pylori* Infection

**Figure 1:**
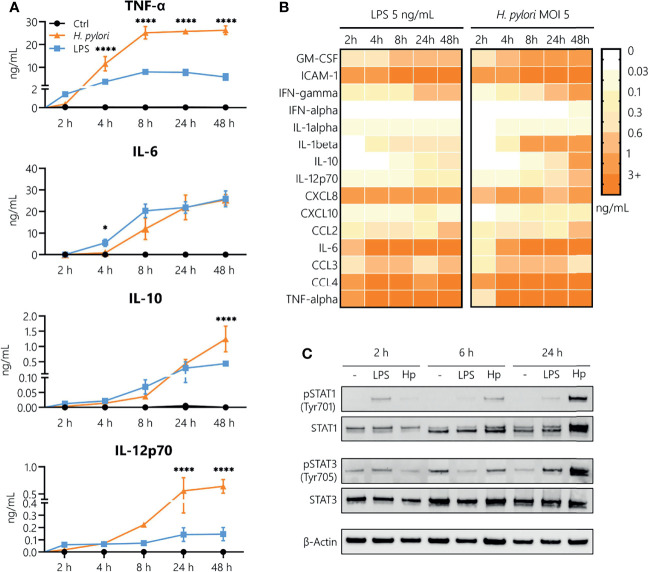
Kinetics of *H. pylori*-induced cytokine and chemokine secretion in human monocytes. **(A)** Human CD14^+^ monocytes were stimulated with LPS (5 ng/mL, blue) or infected with *H. pylori* at MOI 5 (orange), and cytokine secretion was assayed 2, 4, 8, 24 and 48 h post-stimulation. Symbols represent mean ± SD of data from four individual donors. For statistical analysis, two-way ANOVA was performed comparing LPS-treated to *H. pylori*-infected samples at each time point (*p ≤ 0.05, ****p ≤ 0.0001). **(B)** Monocytes were stimulated with LPS (5 ng/mL) or infected with *H. pylori* (MOI 5), and cytokine and chemokine secretion was monitored for the indicated time points. Each value represents the mean of four donors. **(C)** Monocytes were stimulated with LPS (5 ng/mL) or infected with *H. pylori* (MOI 5) for 2, 6 and 24 h. Protein expression and phosphorylation of STAT1 (Tyr701) and STAT3 (Tyr705) as well as expression of β-actin (loading control) were analyzed by Western blot. One representative donor out of two is shown.

In order to analyze the contribution of innate immune cells to the persistent nature of *H. pylori* infection, we examined the kinetics of *H. pylori*-induced secretion of inflammatory mediators in comparison to the prototypical bacterial stimulus LPS derived from *Escherichia coli (E. coli)*. As monocytes are among the first immune cells to infiltrate the inflamed gastric mucosa upon *H. pylori* infection, we focused our investigations on this cell type. The kinetics of cytokine and chemokine release were monitored for 48 h in LPS-stimulated and *H. pylori*-infected cells (**Figures 1A, B**). While, in general, similar cytokines and chemokines are released in response to both stimuli (**Figure 1B**), we observed time-dependent differences in the secretion levels. Compared to *H. pylori*, LPS stimulated a more abundant production of TNF-α and IL-12 in the first 2 h after stimulation. Moreover, while we did not observe a difference at 2 h (when production is very low), IL-10 and IL-6 secretion was markedly increased at 4 and 8 h post LPS stimulation compared to *H. pylori* infection. Starting at 4 h, but most prominently after 8 h to 48 h of stimulation, *H. pylori*-induced cytokine secretion was significantly higher than that induced by LPS, with the exception of IL-6, which was secreted at similar amounts (**Figure 1A**). As cytokines are potent inducers of JAK/STAT signaling, we monitored STAT activation in response to *H. pylori* infection. Again, LPS seems to be more potent than *H. pylori* in inducing STAT1 and STAT3 phosphorylation at early time points (2 h), while after 24 h, *H. pylori*-infected cells showed increased STAT1 protein and STAT1 and STAT3 phosphorylation levels compared to cells subjected to LPS stimulation (**Figure 1C**). This set of data indicates that *H. pylori* induces cytokine and chemokine release in a delayed fashion, with substantially increased secretion levels at later time points compared to LPS stimulation.

### Repeated Exposure to *H. pylori* Results in Tolerance

**Figure 2:**
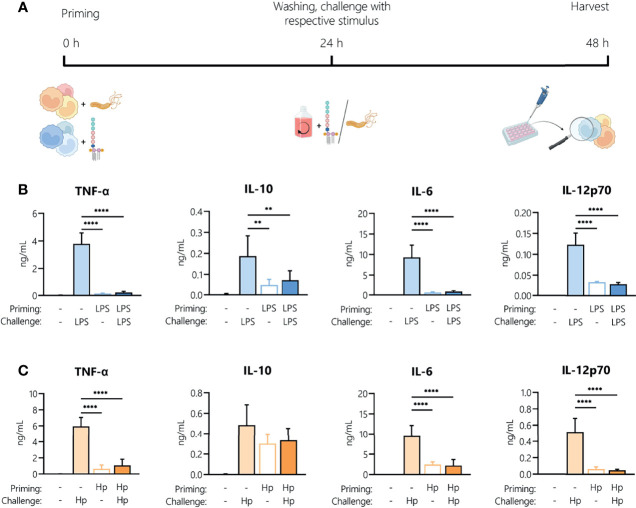
Repeated exposure to *H. pylori* induces tolerance in human monocytes. **(A)** Experimental setup of the monocyte short-term innate response model. Human monocytes were treated with LPS (5 ng/mL, blue bars) or with *H. pylori* (MOI 5, orange bars) for 24 h, washed and re-exposed to the respective stimulus (10 ng/mL LPS, for an additional 24 h. **(B, C)** Cytokine and chemokine secretion by monocytes experiencing two consecutive LPS stimuli **(B)** or *H. pylori* infections (Hp) **(C)** was analyzed by Multiplex assay. Bars indicate mean + SD of four individual donors. For statistical analysis, RM-ANOVA with Šidak’s *post-hoc* test was performed (**p ≤ 0.01, ****p ≤ 0.0001).

It is well established that LPS treatment makes immune cells less responsive to a second LPS stimulus. This can be due to early refractoriness of cells, *e.g.*, ligand-induced receptor down-regulation, or to prolonged tolerance caused by epigenetic and/or metabolic reprogramming (27). This limitation of innate/inflammatory responses induced by bacterial components such as LPS (the aforementioned LPS tolerance) is a key mechanism in maintaining homeostasis and preventing destructive reactivity to repeated challenges against the host. Thus, we aimed to identify how an initial *H. pylori* infection (“priming”) affects the ability of monocytes to respond to a second *H. pylori* exposure (“challenge”). Monocytes were activated with LPS (5 ng/mL) or *H. pylori* (MOI 5) for 24 h then washed and challenged with the same stimulus (filled bars) or left untreated (empty bars) for an additional 24 h (**Figure 2A**). As expected, LPS challenge of unprimed cells (light blue bars, **Figure 2B**) resulted in the secretion of all tested mediators, while challenge of LPS-primed cells (dark blue bars, **Figure 2B**) resulted in a drastic decrease in the production of all cytokines and chemokines examined (**Figure 2B**). LPS-primed cells challenged with medium only (empty bars, **Figure 2B**) still secreted low but measurable amounts of IL-10 and IL-12, suggesting that the cells did not completely return to quiescence. *H. pylori* challenge of unprimed (light orange, **Figure 2C**) cells induced the release of high amounts of all tested mediators (**Figure 2C**). The cytokine release was significantly decreased upon repeated infection with *H. pylori* (dark orange, **Figure 2C**), to the levels observed in unchallenged *H. pylori-*primed cells (empty bars, **Figure 2C**). Notably, IL-10 levels remained high in cells previously infected with *H. pylori*, but again did not increase with re-infection. These data show that *H. pylori* infection results in general tolerance to a second *H. pylori* encounter.

### Priming With *H. pylori* Boosts LPS-Induced Monocyte Activation

**Figure 3:**
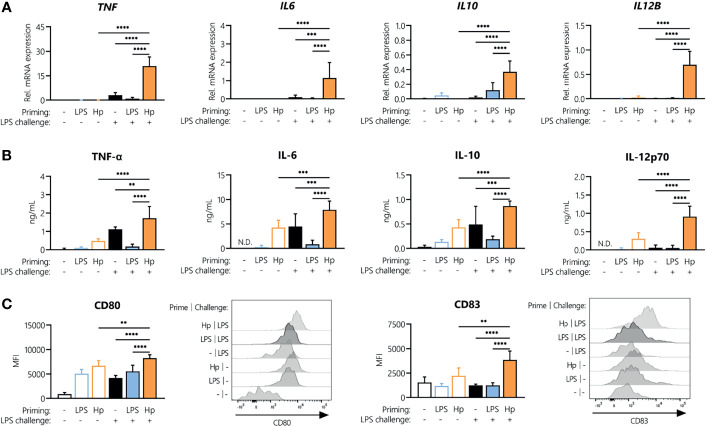
*H. pylori* priming boosts subsequent response to LPS. Human monocytes were primed with either LPS (5 ng/mL) or *H. pylori* (MOI 5) for 24 h and then challenged with LPS (10 ng/mL). **(A)** Cytokine mRNA expression was analyzed 2 h post-challenge. **(B, C)** Cytokine secretion levels as well as surface marker expression were measured after 24 h *via* Multiplex assay or flow cytometry, respectively. Histograms of one representative donor are shown. Bars represent mean + SD of at least four individual donors. For statistical analysis, RM-ANOVA with Šidak’s *post-hoc* test was performed (**p ≤ 0.01, ***p ≤ 0.001, ****p ≤ 0.0001).

As we observed tolerance to repeated *H. pylori* exposure, we next aimed to identify whether *H. pylori*-infected cells can still be activated by a different stimulus, *i.e.*, *E. coli* LPS, or whether *H. pylori* infection could induce cross-tolerance to LPS, as observed for other Toll-like receptor (TLR) ligands (30, 31). To compare the responsiveness of *H. pylori-*primed monocytes to LPS-primed cells, we stimulated monocytes with either *H. pylori* or LPS for 24 h, washed the cells, and then analyzed cytokine mRNA expression as well as the secretion upon a subsequent LPS challenge. As a challenge control, we also monitored cytokine production by primed monocytes exposed to culture medium only (empty bars, **Figure 3**). In the absence of challenge, the mRNA expression of *TNF, IL6, IL10* or *IL12B* was practically undetectable in both LPS- and *H. pylori-*primed monocytes (empty bars, **Figure 3A**). However, as already observed, cells primed with *H. pylori* still secreted appreciable amounts of cytokines even after a washing step and in the absence of an LPS challenge (empty bars, **Figure 3B**). Strikingly, while repeated LPS treatment made the cells unresponsive (**Figures 3A, B**, blue bars), *H. pylori*-primed cells not only retained responsiveness to an LPS challenge, but they showed significantly enhanced expression and secretion of the cytokines TNF-α, IL-6, IL-10 and IL-12 (**Figures 3A, B**, orange bars), compared to unprimed monocytes (black bars, **Figures 3A, B**) and unchallenged cells (empty bars, **Figures 3A, B**). Similarly, the co-stimulatory molecules CD80 and CD83 were expressed at a significantly higher level in *H. pylori-*primed monocytes (orange bars) compared to unprimed monocytes (black bars) in response to an LPS challenge (**Figure 3C**). Notably, the reverse sequence of stimuli, i.e., LPS priming followed by a subsequent *H. pylori* challenge, did not result in increased cytokine secretion compared to cells that were challenged with *H. pylori* only (**Supplementary Figure 1**).

### Active Infection Is Required for Potentiating Monocyte Responsiveness to LPS

**Figure 4:**
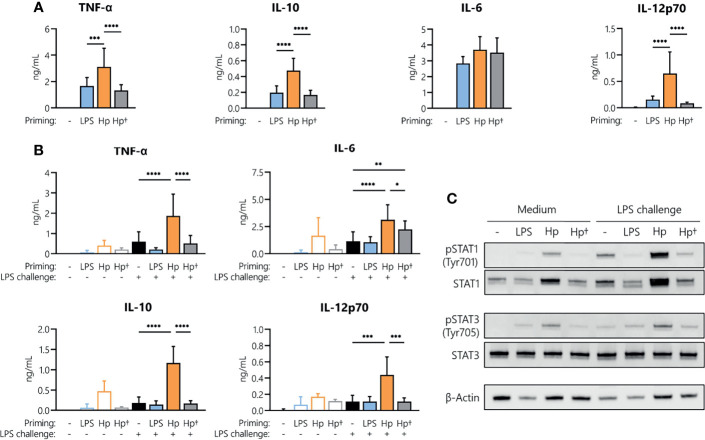
*H. pylori*-induced boost of the monocyte response to LPS requires viable bacteria. Human monocytes were primed with either LPS (5 ng/mL), viable (Hp) or heat-killed (Hp^†^) *H. pylori* (MOI 5) for 24 h and challenged with LPS (10 ng/mL) for an additional 24 h. **(A)** Cytokine secretion 24 h post-priming with respective stimuli. **(B)** Cells primed for 24 h with the indicated stimuli were washed, and cytokine secretion was analyzed 24 h after LPS challenge *via* Multiplex assay. Bars represent mean + SD of three to eight individual donors. For statistical analysis, RM-ANOVA with Šidak’s *post-hoc* test was performed (*p ≤ 0.05, **p ≤ 0.01, ***p ≤ 0.001, ****p ≤ 0.0001). **(C)** Human monocytes were primed with either LPS (5 ng/mL), viable (Hp) or heat-killed (Hp^†^) for 24 h and challenged with LPS (10 ng/mL) for 2 h. Protein phosphorylation and expression of STAT1 (Tyr701) and STAT3 (Tyr705) as well as expression of β-actin (loading control) were analyzed by Western blot. One representative donor out of two is shown.

To understand whether active infection of monocytes with *H. pylori* is essential to prime cells for a potentiated secondary response to other bacterial stimuli, we stimulated monocytes with either viable or heat-killed *H. pylori* and analyzed cytokine secretion after 24 h (**Figure 4A**). Priming with heat-killed bacteria for 24 h resulted in the secretion of TNF-α, IL-6, IL-10 and IL-12 at levels comparable to those induced by LPS, but significantly lower than those induced by viable bacteria (except in the case of IL-6, which was similarly induced by all three stimuli) (**Figure 4A**). We then investigated whether the heat-killed *H. pylori* was still capable of priming monocytes toward an enhanced response to an LPS challenge. Again, monocytes were subjected to priming with viable or heat-killed bacteria, washed after 24 h and challenged with medium alone (control) or containing LPS. While *H. pylori* priming again resulted in a significant increase in cytokine secretion upon LPS challenge (orange bars, **Figure 4B**), priming with heat-killed bacteria (gray bars, **Figure 4B**) did not result in a change of responsiveness to LPS compared to unprimed cells (black bars, **Figure 4B**), except in the case of IL-6, in which priming with killed bacteria significantly enhanced the response to LPS. Accordingly, analyses of STAT1 and STAT3 revealed that phosphorylation of STAT1 in particular and to a lesser extent STAT3 was strongly enhanced in *H. pylori*-primed monocytes upon LPS challenge, while priming of monocytes with heat-killed *H. pylori* did not induce similar effects (**Figure 4C**). Taken together, our data suggest that monocyte responsiveness to LPS can be significantly enhanced by a 24 h pre-exposure to viable *H. pylori*, while heat-killed bacteria have little or no effect.

### *H. pylori*-Induced Innate Memory

**Figure 5:**
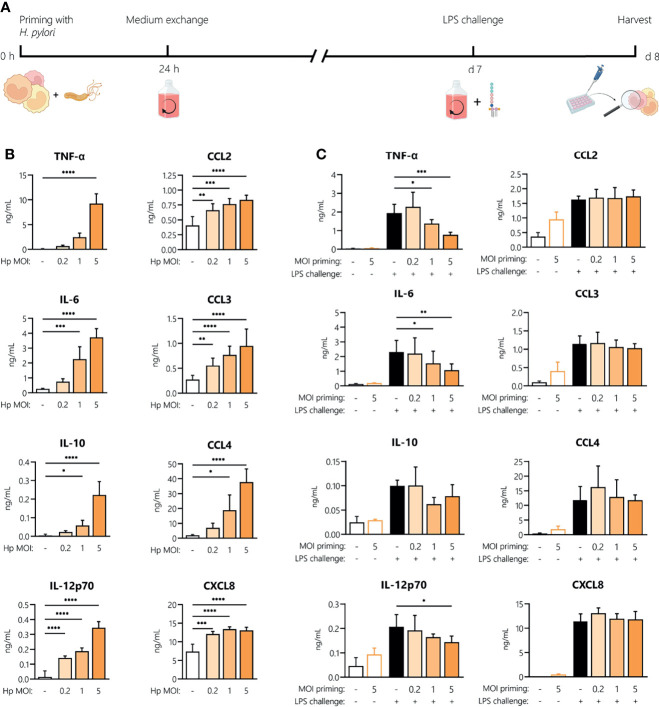
Innate memory response induced by *H. pylori* in monocytes. **(A)** Experimental setup of the monocyte innate memory model. Human monocytes were infected with *H. pylori* (Hp) at the indicated MOI for 24 h, then washed and incubated with fresh medium containing antibiotics for 6 days. Medium was then replaced and cells were challenged with LPS (5 ng/mL) for 24 h. **(B)** Cytokine and chemokine secretion by human monocytes analyzed 24 h post-infection with *H. pylori* at MOIs 0.2, 1 and 5 by Multiplex assay. **(C)** After six days of resting, monocytes were either left untreated (empty bars) or challenged with LPS (10 ng/mL; filled bars). Cytokine and chemokine secretion was measured 24 h post-challenge *via* Multiplex assay. Bars represent mean + SD of at least four individual donors. For statistical analysis, RM-ANOVA with Dunnet’s *post-hoc* test **(B)** and Šidak’s *post-hoc* **(C)** test were performed (*p ≤ 0.05, **p ≤ 0.01, ***p ≤ 0.001, ****p ≤ 0.0001).

To investigate whether *H. pylori* can induce innate memory, we set up an *in vitro* model of innate memory as described previously (29) (**Figure 5A**). Monocytes were infected for 24 h with *H. pylori* at the indicated MOIs, then washed and rested for 6 days, and eventually challenged with LPS for 24 h. As shown in **Figure 5B**, infection with *H. pylori* resulted in a dose-dependent increase of cytokine and chemokine secretion after 24 h. After the resting period, monocyte activation subsided, as shown by the comparable cytokine/chemokine levels between primed cells challenged with medium alone (empty bars, **Figure 5C**) and unprimed unchallenged controls. Challenge with LPS induced a tolerance-type memory response, most evident in cells primed with the highest *H. pylori* MOI and only for inflammatory cytokines (TNF-α, IL-6 and IL-12), not for anti-inflammatory IL-10 or for chemokines.

## Discussion

The well-established concept of endotoxin tolerance describes a negative feedback response to repeated infection with gram-negative bacteria which aims to protect the host while avoiding significant tissue and organ damage triggered by a full-blown immune response. Yet, stimulation of innate immune cells with microbial agents can also have the opposite effect, i.e., it can enhance the responsiveness to subsequent triggers (32, 33). Non-physiological and uncontrolled inflammatory processes are hallmarks of diseases caused by *H. pylori*, such as chronic gastritis or gastric ulcers, and decisively increase the risk of gastric cancer. Since mononuclear phagocytes, including monocytes, directly interact with the pathogen, these cells contribute significantly to the inflammatory environment by secreting cytokines and chemokines, as detailed previously (34, 35). However, the effect of *H. pylori* infection on monocytes and their intrinsic capacity to respond to subsequent inflammatory stimuli remains largely unexplored. Thus, in this study we aimed to describe the functional phenotype of *H. pylori*-infected human monocytes and their responsiveness to an ensuing bacterial stimulus.

Firstly, we established that both LPS and *H. pylori* potently activate primary human monocytes and elicit production of a variety of inflammatory mediators at comparable levels. *H. pylori* infection, though it induced a delayed cytokine release compared to LPS, resulted in a substantial accumulation of inflammatory mediators after 48 h, associated with sustained STAT1 and STAT3 phosphorylation. Moreover, our data show that monocytes infected with *H. pylori* release lower levels of soluble mediators upon a second exposure to the bacterium at close range, suggesting induction of a tolerant phenotype. This is in accordance with literature reporting that a variety of gram-negative bacteria-derived PAMPs and TLR ligands induce immune tolerance (36, 37).

*H. pylori* infection significantly enhanced monocyte responses to a subsequent unrelated bacterial stimulus, (in this case *E. coli* LPS) at the levels of cytokine mRNA expression, polypeptide secretion and surface expression of costimulatory molecules. Notably, only pre-exposure to viable *H. pylori* can induce this hyperactivation of monocytes upon subsequent exposure to LPS, whereas heat-killed *H. pylori* cannot, implying that active infection (rather than TLR engagement by surface bacterial molecules) is responsible for boosting monocyte responses. Additionally, our data reveal that *H. pylori* infection and ensuing LPS challenge result not only in increased cytokine secretion, but also in enhanced STAT1 and STAT3 activation. While STAT3 activation has been observed in epithelial cells (38, 39) as well as immune cells (18, 24, 40) upon *H. pylori* infection, information on involvement of STAT1 is scarce. However, it is well established that interferon gamma (IFNγ), a well-described activator of the JAK/STAT pathway, and TLR signaling act synergistically throughout the course of innate immune responses (41, 42). Studies by Ivashkiv and colleagues revealed that IFNγ enhances a subsequent LPS response in human macrophages by increasing STAT1 occupancy at the *TNF*, *IL6* and *IL12B* loci, indicating that STAT signaling is essential in priming promoters and enhancers to drive LPS-induced hyperactivation of inflammatory genes (42). Intriguingly, in a follow-up study, the same research group showed that IFN not only promotes superinduction of inflammatory genes *via* STAT1 but also selectively suppresses target enhancers that bind STAT3 (41). Moreover, in a recent publication, non-canonical STAT1 phosphorylation has been implicated in the induction of pro-inflammatory cytokine secretion in response to LPS (43), which further emphasizes the role of STAT1 during *H. pylori*-induced innate immune responses.

Immunologic memory is a trait traditionally associated with the adaptive branch of the immune system (44); however, the concept of innate immune memory has attracted significant interest in recent years. This phenomenon describes an altered reactivity of innate immune cells that have previously been exposed to various stimuli and involves both suppressing and potentiating effects (36). In order to test the concept of innate memory in this context and to see if the enhanced state of activation persists, we ‘rested’ *H. pylori*-infected monocytes in culture for 6 days to allow complete extinction of their activation state (the typical model for mammalian innate memory). Intriguingly, the enhancing effect of *H. pylori* infection on the ability of monocytes to respond to LPS is only evident when exposure to LPS takes place shortly after infection (24 h), whereas monocytes infected with *H. pylori* seem less responsive in the innate memory model. In line with this, previous studies using a similar long-term *in vitro* model showed that human monocytes primed with heat-killed *Klebsiella pneumoniae*, *Escherichia coli, Staphylococcus aureus* or *Lactobacillus acidophilus* are less responsive to subsequent LPS or homologous challenge (29, 45, 46). This decrease in mediator production to challenge after a return to baseline activation status implies there are re-programming mechanisms (mostly epigenetic and metabolic changes) tasked with making cells less reactive and therefore less prone to induce tissue damage when faced with temporally distant stimuli.

Collectively, our data indicate that only monocytes which are 1) still in a state of activation due to infection with viable *H. pylori* and 2) capable of producing measurable amounts of inflammation-related cytokines, can react with a heightened response to additional LPS stimulation. This scenario resembles the sustained unique response observed in invertebrates, in which a previously activated immune response is further increased by a second stimulation in order to attain maximal defensive capacity against close-range repeated challenges (47, 48). This pronounced monocyte activation is probably the most effective way to fight off a threatening pathogen. However, because the stomach is not a sterile organ, this hyper-responsiveness to bacterial PAMPs could result in inflammation-inflicted tissue damage. Accordingly, recent advances identified a wide variety of bacterial species native to the gastric environment, including *Streptococcus* and *Staphylococcus* (49), emphasizing the need for studying the potential of *H. pylori*-primed monocytes to react to antigens derived from these gastric bacteria. The presence of a diverse microbiota in the gastric compartment could increase the likelihood of *H. pylori*-primed monocytes encountering other bacterial antigens, which could result in hyper-activation. In support of this, it was documented that a lack of commensal flora in *H. pylori*-infected mice reduced signs of gastritis and delayed gastric neoplasm formation (50, 51).

Thus, we propose that during chronic *H. pylori* infection, monocytes can become hyper-responsive to bacterial agents (PAMPs), thereby contributing to the inflammatory environment and leading to progressive pathological tissue damage in the gastric lining, including gastritis, ulceration and stomach cancer.

## Data Availability Statement

The raw data supporting the conclusions of this article will be made available by the authors, without undue reservation.

## Author Contributions

TF and TN contributed equally to this work TF, TN, and MM performed the research, prepared the figures and wrote the manuscript. H-HD contributed to the experiments. DB and JH-H wrote the manuscript and contributed to the experimental design. JH-H supervised the work. All authors contributed to the article and approved the submitted version.

## Funding

This work was supported by the Austrian Science Fund (FWF) [grant number P29941], the County of Salzburg, Cancer Cluster Salzburg [grant number 20102-P1601064-FPR01-2017], and by the Priority program ACBN, University of Salzburg.

## Conflict of Interest

The authors declare that the research was conducted in the absence of any commercial or financial relationships that could be construed as a potential conflict of interest.

## Publisher’s Note

All claims expressed in this article are solely those of the authors and do not necessarily represent those of their affiliated organizations, or those of the publisher, the editors and the reviewers. Any product that may be evaluated in this article, or claim that may be made by its manufacturer, is not guaranteed or endorsed by the publisher.
