# Supplementary figures and images for: Helicobacter pylori Infection of Primary Human Monocytes Boosts Subsequent Immune Responses to LPS
Source: Front Immunol. 2022 Mar 2;13:847958. doi: 10.3389/fimmu.2022.847958 (PMC8924073; doi:10.3389/fimmu.2022.847958)

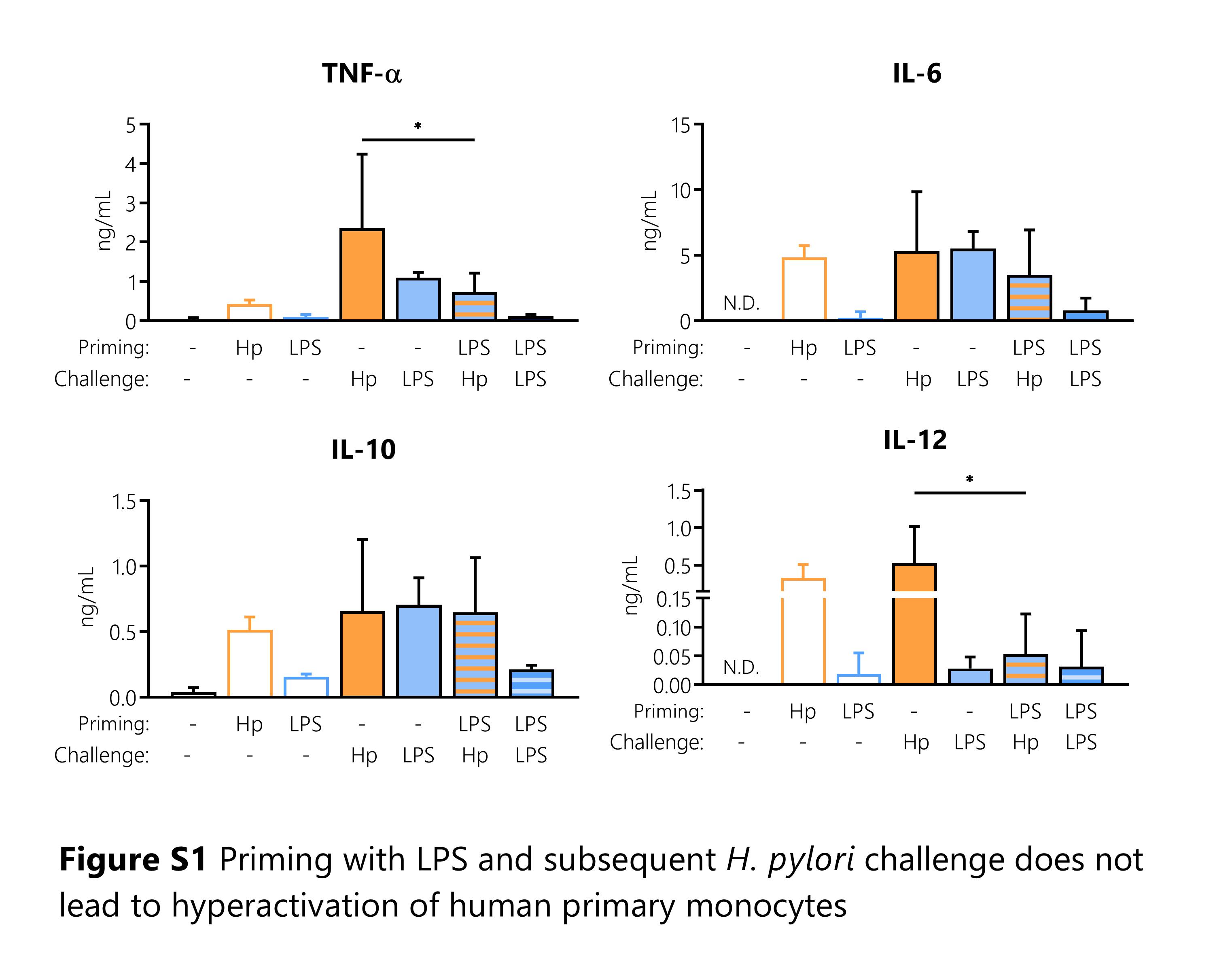

Supplement: Supplementary file 1 [file Image_1.jpeg]
